# Orocervical Actinomycosis in a Patient With Neck Swelling and Occipital Headache: A Case Report

**DOI:** 10.1155/crdi/4580275

**Published:** 2025-12-26

**Authors:** Mehran Frouzanian, Ahmad Alikhani, Amirsaleh Abdollahi

**Affiliations:** ^1^ Department of Infectious Diseases, School of Medicine, Mazandaran University of Medical Sciences, Sari, Iran, mazums.ac.ir; ^2^ Infectious Diseases Department and Antimicrobial Resistance Research Center and Transmissible Diseases Institute, Mazandaran University of Medical Sciences, Sari, Iran, mazums.ac.ir

**Keywords:** actinomyces, actinomycosis, cervicofacial actinomycosis, skull base infections

## Abstract

Actinomycosis is a rare bacterial infection caused by *Actinomyces* species, most commonly affecting the cervicofacial region. Posterior skull base involvement is extremely uncommon. We report a case of actinomycosis in a 44‐year‐old male with a 10‐year history of chronic hepatitis B on tenofovir therapy, who presented with a 3‐month history of progressive right‐sided neck swelling, pain, occipital headaches, and a 10 kg weight loss. The patient initially experienced a sore throat and neck discomfort, partially relieved by medication, but gradually developed a foreign body sensation in the retropharynx and painful swelling in the lateral neck and behind the right ear, accompanied by fever and chills. Physical examination revealed swelling, erythema, warmth over the right lateral neck, and neck stiffness. MRI showed an ill‐defined infiltrative lesion at the posterior skull base, involving the right occipital bone and upper cervical vertebrae, extending into adjacent soft tissues. Biopsy of the lesion demonstrated filamentous organisms with sulfur granules amid a mixed inflammatory response, confirming actinomycosis. The patient was treated with intravenous ceftriaxone followed by oral amoxicillin, along with surgical drainage from the right posterior cervical region. At the 11 week follow‐up, he showed significant clinical improvement, with continued long‐term antibiotics due to bone involvement. This case highlights the diagnostic challenges posed by atypical presentations of actinomycosis, particularly in patients with predisposing conditions such as chronic infections. Unlike typical cervicofacial actinomycosis, this patient lacked classical features such as discharging sinuses or lymphadenopathy. Recognition of sulfur granules in biopsy specimens was pivotal for diagnosis. Early diagnosis, prolonged high‐dose antibiotic therapy, and surgical intervention when needed are crucial to achieving favorable outcomes. Clinicians should maintain a high index of suspicion for actinomycosis in patients presenting with unexplained neck masses and skull base lesions.

## 1. Introduction

Orocervical actinomycosis is a rare infection that poses significant diagnostic challenges due to its tendency to mimic malignancies or granulomatous lesions [1]. *Actinomyces* species are naturally found in the oral cavity, gastrointestinal tract, and pelvic regions but do not exist freely in the environment [2]. Cervicofacial, thoracic, and abdominal actinomycosis are the most common types. Central nervous system actinomycosis is rare and typically occurs due to direct extension from an adjacent focus or through hematogenous spread from a distant site [1, 3]. Diagnosis is further complicated by the disease’s limited recognition and the difficulty in culturing *Actinomyces* because of its fastidious nature [4–6]. Treatment is complex, as the infection causes extensive tissue destruction. Patients with actinomycosis require extended high‐dose treatment (6–12 months) with penicillin G or amoxicillin to ensure effective drug penetration into abscesses and infected tissues [5, 7, 8]. However, if optimal surgical removal of infected tissues is achieved, the antimicrobial therapy duration may be reduced to approximately 3 months [7]. Surgical management alone is often associated with a high rate of recurrence, but combining surgery with short‐term antibiotic therapy has yielded good outcomes. However, surgery carries risks, especially in cases of extensive lesions [5]. Therefore, treatment plans should be customized based on the location, extent of infection, and the patient’s response to antibiotic therapy.

## 2. Case Presentation

A 44‐year‐old male, with a known case of hepatitis B for the past 10 years, presented with a three‐month history of progressively worsening right‐sided neck pain and swelling. Initially, he developed a sore throat and diffuses anterior neck discomfort, which caused difficulty in swallowing and mild limitation in neck movement. These symptoms partially subsided with medication but did not completely resolve. Over the next several weeks, the patient noticed gradually increasing swelling and tenderness along the right lateral neck, which later extended toward the posterior cervical (suboccipital) region. Approximately 2 weeks before admission, his symptoms acutely worsened, with the onset of a painful, firm swelling over the right lateral and posterior cervical region, radiating toward the area behind the right ear. He also reported a persistent sensation of fullness or obstruction in the retropharyngeal area, particularly during swallowing. There was no history of hearing loss, ear discharge, or otalgia. During the early phase of illness, he experienced low‐grade fever and chills, which resolved spontaneously. Over the 3‐month period, the patient reported a 10 kg unintentional weight loss and progressive fatigue associated with neck pain and stiffness.

The patient has a past medical history of chronic hepatitis B, diagnosed 10 years ago, and is currently taking tenofovir 300 mg daily as part of his treatment. There is no significant family history or social risk factors contributing to his current condition.

On physical examination, the patient appeared ill but not toxic. He was alert, oriented, and able to respond appropriately to questions. His vital signs were stable, with a blood pressure of 120/75 mmHg, pulse rate of 80 bpm, respiratory rate of 16 breaths per minute, and a temperature of 36.8°C. General examination revealed no petechiae, purpura, or ecchymosis. Examination of the head showed no deformities or signs of trauma. The external auditory canals were clear, and the tympanic membranes were intact, pearly gray, and mobile on pneumatic otoscopy, without evidence of effusion, retraction, or perforation. The eyes appeared normal, with no pallor of the conjunctiva, no icterus of the sclera, and no signs of conjunctivitis. There were no abnormalities in the nose, with no rhinorrhea, epistaxis, or nasal congestion. The oral cavity was unremarkable except for multiple decayed teeth; the mucosa was of normal color, and there were no gingival bleeding, ulcers, or aphthous lesions. Examination of the throat revealed no erythema, exudates, tonsillar hypertrophy, or deviation of the uvula. However, a retropharyngeal bulge was noted on the posterior pharyngeal wall extending to right anterior part, causing mild narrowing of the oropharyngeal airway and posterior displacement of the uvula. The overlying mucosa appeared intact and non‐fluctuant, suggesting a deep‐space process involving the prevertebral or parapharyngeal tissues rather than a superficial abscess.

On general physical examination, the patient appeared moderately ill but was hemodynamically stable and in no acute distress. His posture was slightly guarded due to neck discomfort. There was no pallor, cyanosis, clubbing, or peripheral edema. No petechiae or ecchymoses were observed on the skin. Examination of the neck revealed a tender, firm swelling involving the right lateral and posterior cervical regions, extending from just below the mastoid tip and postauricular area inferiorly to the level of the C3–C4 vertebrae, and anteromedially toward the upper sternocleidomastoid region. The overlying skin was erythematous and warm to touch, and neck movements were markedly restricted, especially on rotation and extension. The swelling was nonfluctuant, and there was no overlying sinus tract or discharge. The trachea was midline, and there was no jugular venous distention. No cervical, axillary, or inguinal lymphadenopathy was palpable. Systemic examination revealed a symmetrical chest with normal respiratory excursions and no spinal deformities such as kyphosis, scoliosis, or lordosis. Cardiac auscultation showed normal S1 and S2 sounds without murmurs. Respiratory examination was unremarkable, with vesicular breath sounds and no adventitious sounds. The abdomen was soft, nontender, and without organomegaly, and bowel sounds were normal. The patient was alert and oriented to time, place, and person, and cranial nerves II–XII were intact. A detailed neurological assessment was partially limited due to neck stiffness, though deep tendon reflexes were 2+ bilaterally. The extremities showed no cyanosis or edema, and capillary refill time was normal.

The patient’s laboratory investigations revealed a white blood cell count of 9200 cells/μL, with 75.5% neutrophils and 14% lymphocytes. Hemoglobin was measured at 10.3 g/dL, and the platelet count was 336,000/μL. His coagulation profile showed a prothrombin time of 13.5 s and an INR of 1. The biochemical profile indicated serum sodium, potassium, blood glucose, blood urea nitrogen, and creatinine in normal ranges.

**Figure 1:**
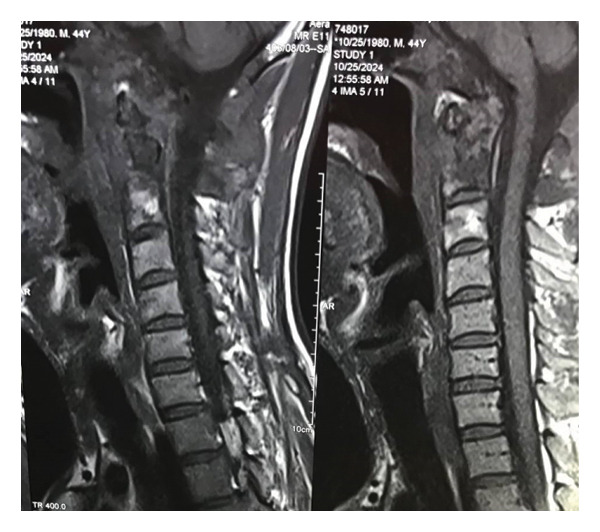
MRI of the patient showed an infiltrative enhancing lesion at the posterior skull base, affecting the right occipital bone, C1, and C2 vertebrae, with extension into the prevertebral and parapharyngeal spaces.

A contrast‐enhanced magnetic resonance imaging (MRI) scan of the head and neck was performed to evaluate the extent of the lesion. The MRI revealed an ill‐defined, infiltrative, contrast‐enhancing lesion centered at the posterior skull base, primarily involving the right occipital bone and extending inferiorly to involve the atlas (C1) and axis (C2) vertebral bodies. The lesion demonstrated extension into the adjacent prevertebral and right parapharyngeal spaces, with infiltration of the right posterior upper cervical musculature, including the suboccipital and paraspinal muscles. The radiological impression suggested a chronic infiltrative process, with a differential diagnosis of infection (such as actinomycosis) versus an infiltrative neoplasm. Additionally, mild fluid signal intensity within the right mastoid air cells indicated possible early mastoiditis, and a retrocerebellar arachnoid cyst measuring 35 × 17 mm was incidentally noted in the posterior cranial fossa. The remaining brain parenchyma, brainstem, and pituitary gland appeared unremarkable (Figure 1).

A biopsy was performed from the soft tissue lesion on the right side of the foramen magnum using a percutaneous excisional approach. The specimen consisted of two cream‐colored cylindrical fragments, each measuring 1.5 × 0.2 × 0.2 cm, with full saturation (100%) and documented orientation of 2/1. Histopathological examination revealed fibromuscular tissue extensively infiltrated by a lymphohistiocytic inflammatory infiltrate. Slender filamentous organisms surrounded by eosinophilic proteinaceous material (sulfur granules) were identified. A mixed inflammatory response comprising neutrophils, plasma cells, eosinophils, and lymphocytes was present, along with scattered multinucleated giant cells and noncaseating granulomas. These findings are consistent with an inflammatory process suggestive of actinomycosis.

Penicillin, the first‐line therapy, was unavailable at the hospital; therefore, the patient received ceftriaxone 1 g intravenously every 12 h for 3 weeks during hospitalization, followed by oral amoxicillin for 8 weeks postdischarge. Surgical drainage of the lesion was performed via a posterior neck approach, behind the right ear, under general anesthesia. At the time of the biopsy, the patient was receiving empiric antibiotic therapy. At the 11‐week follow‐up, continuation of antibiotic therapy was advised. Clinical symptoms, including neck pain, movement restriction, retropharyngeal swelling, and erythema, had resolved, and MRI demonstrated a reduction in the size of the lesion. Due to associated bone involvement, long‐term antibiotic therapy is ongoing.

## 3. Discussion

Actinomycosis is often the most misdiagnosed disease due to its diagnostic challenges. The condition is primarily caused by the gram‐positive bacterium *Actinomyces israelii*. Less frequently, other species such as the *A. naeslundii/viscosus* complex, *A. odontolyticus*, *A. meyeri*, and *A. gerencseriae* can also be responsible for the disease [7].

A key feature of actinomycosis is the presence of “sulfur granules” in aspirated material, which are lobulated microcolonies of *Actinomyces* observed microscopically in biopsy samples. Medical management primarily involves penicillin, as these bacteria are highly susceptible to beta‐lactam antibiotics [9, 10]. While other antibiotics like macrolides and tetracyclines can be used, they may not provide significant benefits. Pioneering studies have suggested prolonged high‐dose penicillin therapy for effective treatment, with some advocating for surgical removal of infected tissue alongside antibiotic therapy for improved outcomes.

Actinomycosis is an opportunistic infection primarily affecting the oral cavity, with the tonsils and decayed teeth being the most common sites. *Actinomyces* are filamentous, nonspore‐forming bacteria, thrive in low‐oxygen environments and are naturally found in gingival crevices, periodontal pockets, and decayed teeth [7]. The three main forms of actinomycosis include cervicofacial, pulmonary, and gastrointestinal types. Notably, a retrospective study from the University of Cologne found that actinomycotic infections primarily affect the mandible (53.6%), cheek (16.4%), and chin (13.3%), among other sites [5, 10].

While cervicofacial actinomycosis has been well documented, occurrences in the submandibular gland are exceptionally rare, often presenting without identifiable predisposing factors [11]. However, approximately half of the patients with actinomycosis reported a history of local trauma causing mucosal damage, suggesting that injury to healthy tissue can be a predisposing factor. In our case, no foreign body was found within the gland; instead, the swelling followed an injury, highlighting that actinomycosis can arise from mucosal breakdown, which is essential for infection, as *Actinomyces* do not invade healthy tissue [12].

Typically, cases exhibit discharging sinuses, hyperemia, fever, and tenderness, but surprisingly, none of these features were present here, raising suspicion for a malignant cause. In the context of actinomycosis, the differential diagnosis of posterior neck masses is essential, as it can mimic other conditions, including malignancies and various infections. The clinical presentation of actinomycosis in the neck region, such as swelling and pain, may overlap with that of more serious diseases, including tumors. For instance, a large, aggressive mass without lymphadenopathy can raise suspicion for malignancy, especially when inflammatory signs such as fever and discharging sinuses are absent. Additionally, other infections, like tuberculosis, deep neck space infections, and fungal infections, may present with similar clinical features. Therefore, distinguishing actinomycosis from these conditions requires careful consideration of the patient’s history, imaging findings, and microbiological studies. The absence of typical signs of malignancy, such as well‐defined margins on imaging or the presence of significant inflammatory changes, can help guide the diagnosis towards actinomycosis, which often presents with nonspecific imaging results. A thorough differential diagnosis is crucial in ensuring that appropriate treatment, such as prolonged antibiotic therapy, is administered promptly, avoiding misdiagnosis and unnecessary interventions.

The absence of lymphadenopathy alongside a large, aggressive mass can help differentiate orocervical actinomycosis from malignancy, as the latter usually has well‐defined margins and does not cause significant inflammatory changes in surrounding soft tissue. Diagnostic imaging techniques, such as contrast‐enhanced CT scans, may aid in the diagnosis, though they often yield nonspecific results [7].

Previous case reports have predominantly described cervicofacial actinomycosis, which typically presents with mandibular or submandibular swelling, often associated with poor oral hygiene, dental infections, or trauma [13–16]. In contrast, orocervical actinomycosis involving the posterior skull base and suboccipital region—as in this case—is exceptionally uncommon. Unlike classical cervicofacial presentations, which frequently show superficial abscesses, draining sinus tracts, or palpable lymphadenopathy, posterior skull base involvement often manifests with deep‐seated neck swelling, retropharyngeal bulging, and nonspecific symptoms such as occipital headache or neck stiffness, making clinical recognition difficult. The rarity is further compounded by the anatomical complexity of the posterior skull base, where infection may spread insidiously to bone and prevertebral muscles, mimicking neoplastic or granulomatous lesions on imaging. Consequently, this variant is less studied, poorly documented, and often underrecognized, highlighting the diagnostic challenge and the need for histopathological confirmation to guide effective management.

Xu et al. study described cervical actinomycosis with spinal cord compression, presenting as nonspecific back pain and limb weakness, challenging to diagnose due to similarities with metastatic tumors. Surgical debridement and histopathology confirmed actinomycosis, enabling effective treatment with long‐term antibiotics [17]. In our case, the patient presented with progressive neck pain, swelling, and significant weight loss. MRI revealed an infiltrative lesion involving the occipital bone, C1, C2, and parapharyngeal spaces, but without spinal compression. A biopsy confirmed actinomycosis, showing sulfur granules in an inflammatory background. After 7 weeks of antibiotics, our patient’s symptoms and lesion size improved, though bone involvement necessitated continued therapy. Both cases highlight the need for prompt diagnosis and extended antibiotic treatment to prevent severe complications, with ours contributing new insights into nonspinal compressive forms of cervical actinomycosis.

Cervicofacial actinomycosis, while relatively common, can pose serious health risks and result in severe complications, making prompt detection essential. Accurate identification of the disease is critical. A negative bacterial culture does not rule out the condition, and surgical intervention remains critical for both confirming the diagnosis and providing treatment. Studies have shown that removing the affected tissue surgically, followed by an extended regimen of broad‐spectrum antibiotics, can successfully eliminate the infection without lasting effects [7]. Nevertheless, surgery alone frequently leads to recurrence, and extensive tissue removal may cause considerable morbidity. Consequently, a combined strategy of antibiotic therapy and surgical management is recommended for cases that do not respond to medication alone, optimizing patient outcomes.

## 4. Conclusion

Orocervical actinomycosis, though rare, should be considered in the differential diagnosis of neck masses, particularly in patients with underlying conditions. This case demonstrates the complexity of diagnosing actinomycosis due to its atypical presentation, mimicking other serious conditions such as malignancies and infections. Early recognition, thorough appropriate diagnostic work‐up, and treatment with prolonged antibiotics are essential for achieving favorable outcomes. Surgical intervention may also be necessary, especially in cases with significant tissue involvement, as seen in this patient. Timely management can lead to substantial recovery, though ongoing long‐term antibiotic therapy is essential to prevent relapse.

## Ethics Statement

Ethical approval was not required for this case report, as per the policies of the Mazandaran University of Medical Sciences regarding single‐patient case studies. Written informed consent was obtained from the patient for publication of this case and any accompanying clinical information or images. All efforts have been made to ensure the patient’s anonymity and confidentiality.

## Consent

Informed consent was obtained from the patient for both study participation and publication of identifying information/images in an online open‐access publication.

## Conflicts of Interest

The authors declare no conflicts of interest.

## Author Contributions

Ahmad Alikhani oversaw and treated the case, including the entire revision process, and contributed to the article’s composition. Mehran Frouzanian authored the article, including the discussion section, and participated in the complete revision. Amirsaleh Abdollahi played a role in crafting the case report discussion and participated in the entire revision process.

## Funding

This research did not receive any funding or financial support.

## Data Availability

Data sharing is not applicable to this article as no datasets were generated or analyzed during the current study.
